# Integrated biochar and green manure improve maize productivity and soil health under reduced nitrogen fertilization in yellow soil

**DOI:** 10.3389/fpls.2026.1797967

**Published:** 2026-04-15

**Authors:** Quanquan Wei, Pengfei Liu, Xiangyang Guo, Meng Zhang, Lingling Liu, Xiaofeng Gu, Lei Zhang, Yifei Zou, Yu Li, Muzammal Rehman, Jiulan Gou

**Affiliations:** 1 Institute of Soil and Fertilizer, Guizhou Academy of Agricultural Sciences, Guiyang, Guizhou, China; 2 Guizhou Observation Experimental Station of Farmland Preservation and Agricultural Environmental Sciences, Ministry of Agriculture, Guiyang, Guizhou, China; 3 Institute of Upland Crops, Guizhou Academy of Agricultural Sciences, Guiyang, China; 4 MOA Key Laboratory of Crop Ecophysiology and Farming System in the Middle Reaches of the Yangtze River, College of Plant Science and Technology, Huazhong Agricultural University, Wuhan, Hubei, China; 5 Rapeseed Research Institute, Guizhou Academy of Agricultural Sciences, Guiyang, Guizhou, China; 6 Yunnan Key Laboratory of Potato Biology, School of Life Science, Yunnan Normal University, Kunming, China

**Keywords:** biochar, carbon fractions, green manure, soil aggregates, yellow soil

## Abstract

**Introduction:**

Excessive nitrogen use threatens the environmental sustainability and soil health. Application of biochar or green manure is a promising strategy to improve the soil health and plant productivity. However, the effects of their combined application in yellow soils remain underexplored.

**Methods:**

A two-year field study was conducted to investigate the combined effects of biochar and green manure on maize productivity and soil health. The experimental treatments were comprised of zero fertilizer (control), 100% nitrogen fertilizer, 100% nitrogen with biochar, 100% nitrogen with green manure, 100% nitrogen with biochar and green manure, and 80% nitrogen with biochar and green manure. Maize yield and soil properties were measured.

**Results:**

Results showed that plant yield, soil nutrient content, carbon fractions, and enzyme activities were significantly high at 100% nitrogen with biochar and green manure application, compared with control. Furthermore, application of 100% nitrogen with biochar and green manure achieved the highest water-stable macro-aggregates (WS-MA), the smallest percentage of aggregate destruction (PAD), and the optimal maize yield. However, maximum nitrogen-use efficiency was observed at 20% reduced nitrogen with biochar and green manure application. Soil carbon components were significantly positively correlated with water-stable aggregates and enzyme activities, and the soil quality index (SQI) was linearly positively correlated with ecosystem multifunctionality (EMF).

**Conclusions:**

Combined application of biochar and green manure significantly improves soil fertility in yellow soils by enhancing nutrient availability, aggregate stability, carbon fractions, and soil enzyme activities. Notably crop yield and quality were maintained even with a 20% reduction in nitrogen fertilizer. These findings highlight the effectiveness of integrating biochar and green manure as a sustainable fertilization strategy that enhances soil health while reducing chemical nitrogen inputs.

## 1 Introduction

Maize (*Zea mays* L.), known for its drought resistance and tolerance to low soil fertility, is a predominant crop in the mountainous regions of Southwest China (Sichuan, Yunnan, Guizhou, Chongqing, and Guangxi provinces), which may form a significant part of China’s maize belt. However, long-term monoculture and intensive maize cropping systems have led to a series of negative impacts on soil health (Chen et al., 2025), such as significant reductions in organic carbon, nutrient depletion, decreased microbial diversity, and enhanced soil erosion, resulting in a decline in sustainable soil productivity. Enhancing the fertility of these soils is therefore critical for sustainable agriculture. Soil fertility can be evaluated by physicochemical indicators, including soil organic matter content, nutrient levels, pore structure, and aggregate stability (Lallaouna et al., 2025). An appropriate nitrogen level is important for improving crop growth and soil characteristics (Zhang et al., 2026, 2025).

Soil aggregates are key structural units, with their formation and stability shaped by the content and nature of soil organic carbon (Zhou et al., 2020; Liu Y. et al., 2023). These aggregates foster favorable microenvironments that boost microbial biomass and enzyme activity, thereby promoting soil carbon mineralization (Li et al., 2023). Soil quality, a core indicator of the soil’s capacity to sustain environmental, plant, animal, and human health, is evaluated through an integrated system encompassing physical, chemical, and biological characteristics (Lu et al., 2018; Wu et al., 2024). Ecosystem multifunctionality (EMF), which reflects the soil’s capacity to sustain multiple ecological functions simultaneously, is closely linked to enzymatic activities that drive nutrient cycling (Nannipieri et al., 2012; Manning et al., 2018).

Green manure as high-quality carbon and nitrogen sources are widely used to enhance soil fertility. Green manure cultivation and incorporation not only provide substantial mineral nutrients and organic matter (Zhou et al., 2023), but also significantly improve crop yield and quality (Liang et al., 2024) by enhancing soil microbial community structure, diversity (He et al., 2020; Xia et al., 2025), and enzyme activity. This practice also provides concurrent ecological and environmental benefits (Yang et al., 2019; Liang et al., 2023). However, due to its high proportion of labile carbon (Zhu et al., 2014), green manure incorporation results in relatively slow accumulation of soil organic carbon (SOC) (Zhang J. et al., 2023; Zhang X. et al., 2023) and may pose an increased risk of greenhouse gas emissions when co-applied with inorganic fertilizers (Lou et al., 2024). Biochar, a stable carbon source characterized by high pH and a high carbon-to-nitrogen ratio, has significantly greater carbon stability than straw. Its application effectively improves soil aggregate structure (Liu Z. et al., 2023), promotes microbial activity and community abundance (Awad et al., 2017);, and mitigates the inhibition of nitrifying bacterial communities (Cayuela et al., 2024; Hu et al., 2024). Furthermore, by increasing the abundance of functional genes such as amoA, nirK, nirS, and nosZ in acidic yellow soils, biochar significantly reduces N_2_O emissions and NO_3_^-^ leaching (Oladele et al., 2019). However, due to its low microbial bioavailability (Yang et al., 2024; Li et al., 2025), excessive application can adversely affect soil structure and microbial communities (Xiang et al., 2021).

Although the individual application of either biochar or green manure can improve crop yield and soil fertility, each has distinct limitations: green manure has insufficient carbon stability, while biochar has limited microbial bioavailability. Co-incorporation of these two amendments generates a synergistic effect that optimizes the soil carbon-to-nitrogen (C/N) ratio. This synergy enhances organic carbon mineralization concurrently with promoting the formation of stable carbon pools and improving carbon bioavailability, thereby addressing the limitations of their individual application.

Previous studies have primarily focused on the individual application of biochar or green manure (Zhang M. et al., 2023; Zhang J. et al., 2023), elucidating their respective potentials for soil improvement but failing to address the limitations of separate use. Although synergistic application has demonstrated significant advantages in carbon and nitrogen regulation, there remains a lack of systematic research exploring the effects of combined biochar and green manure incorporation on soil nutrients, aggregate structure, carbon fractions, and enzyme activities. This is particularly true for long-term field positioning trial data. To address this gap, present study, utilizing maize cultivated in yellow soil, conducted a two-year consecutive field experiment to investigate the changing patterns of soil nutrients, aggregates, carbon components, and enzyme activities under the combined application of biochar and green manure, as well as its impact on maize yield. Yellow soil is a dominant agricultural soil type in Guizhou province in Southwest China (Lei et al., 2026), comprising approximately 38.7% of the province’s total soil area and accounting for 47.8% of the national yellow soil distribution (Han et al., 2024); It features high clay content, compactness, acidity, and infertility. Therefore, the primary objective of present research was to establish a foundation for improving the quality of yellow soil in Guizhou and reducing the demand for nitrogen fertilizer in maize cultivation, thereby supporting the synergistic development of agricultural ecology and production efficiency in karst mountainous areas.

## 2 Material and methods

### 2.1 Description, materials collection, and treatments

A two-year consecutive field experiment was conducted at the National Soil Quality Guiyang Scientific Observation and Research Station (also the Ministry of Agriculture and Rural Affairs Guizhou Cultivated Land Conservation and Agro-Environment Observation and Research Station), Guizhou Academy of Agricultural Sciences, Guiyang City, Guizhou Province, commencing in October 2022. To prevent interference between experimental plots, impermeable marble partitions (60 cm length × 60 cm width × 10 cm thickness) were installed around each plot. The experimental site experiences a subtropical humid and mild climate. The soil at the experimental site was a typical yellow soil from Guizhou upland, with key physicochemical properties such as pH 6.65, organic matter 44.45 g/kg, total nitrogen 2.00 g/kg, available phosphorus 33.95 mg/kg, and available potassium 219.89 mg/kg. During the experimental period, the mean air temperature was 15.3 °C, the mean annual relative humidity was 77%, and the mean annual precipitation was 1094.1 mm.

In this study, the tall-stature maize (*Zea mays* L.) cultivar Nongfa 710 (sourced from the Upland Crops Research Institute, Guizhou Academy of Agricultural Sciences) served as the experimental crop. Chemical fertilizers comprised urea (46% N), calcium superphosphate (12% P_2_O_5_), and potassium sulfate (50% K_2_O). Biochar was produced from crop straw using pyrolysis equipment supplied by the Henan Biochar Engineering Technology Research Center under limited-oxygen pyrolysis conditions at 380-400 °C for 20 minutes. Common vetch (*Vicia sativa* L.) was used as the green manure.

The experiment comprised six treatments, i.e., (1) no nitrogen application (CK); (2) 100% nitrogen (T1); (3) 100% nitrogen with biochar (T2); (4) 100% nitrogen with green manure (T3); (5) 100% nitrogen with biochar and green manure (T4); and (6) 80% nitrogen with biochar and green manure (T5).

### 2.2 Crop management

Each plot measured 15.675 m^2^ (4.75 ^m^ length × 3.3 m width). Maize seedlings were transplanted at a density of 5 rows per plot, with 17 plants per row, resulting in 85 plants per plot (equivalent to 5.423×10^5^ plants hm^-2^). Total fertilizer application rates during the maize growing season were N (100% application level) at 180 kg hm^-2^, P_2_O_5_ at 120 kg hm^-2^, and K_2_O at 150 kg hm^-2^. Nitrogen fertilizer was split-applied three times: 40% as basal fertilizer, 40% as the first topdressing, and 20% as the second topdressing. Phosphorus (P_2_O_5_) and potassium (K_2_O) fertilizers were applied entirely as basal dressings. Both green manure and biochar were incorporated into the soil 5–10 days prior to maize transplanting. Fresh green manure was applied at a rate of 22500 kg hm^-2^, with an incorporation depth of 20 cm. Biochar, produced entirely from the residual straw harvested from the corresponding plot in the previous season at a rate equivalent to 3000 kg hm^-2^, was applied simultaneously with the green manure as a single application.

### 2.3 Soil sampling

After the two-year experiment, soil samples (0–20 cm depth) were collected from each plot at maize harvest in September 2024 using a five-point sampling method. Samples from each plot were composited, transported to the laboratory, and manually disaggregated along natural fractures. The composited soil was sieved (<10 mm) to remove large debris and stones while preserving natural soil aggregates and minimizing disturbance to soil physical structure and then air-dried in the dark at ambient temperature. After air-drying, any further visible fine root or stone was removed by hand. The soil was quartered into two subsamples. One subsample was ground to pass through 20-mesh (<850 μm) and 100-mesh (<150 μm) sieves for analysis of soil chemical properties, carbon fractions, and enzyme activities. The other sub-sample was used for aggregate stability analysis.

### 2.4 Soil chemical analysis

Soil pH was measured using a pH meter. Organic matter (OM) content was determined by the potassium dichromate volumetric method (Zeyede, 2020). Total nitrogen (TN) content was quantified via semi-micro Kjeldahl digestion (Peng et al., 2019). Available phosphorus (AP) was extracted using sodium bicarbonate and measured by molybdenum-antimony colorimetry (Olsen et al., 1954; Murphy and Riley, 1962). Available potassium (AK) content was determined by flame photometry (Bao, 2000). Total carbon (TC) was determined by mass spectrometry. SOC and readily oxidizable carbon (ROC) were quantified using the potassium dichromate oxidation method. Dissolved organic carbon (DOC) was extracted with deionized water and measured by mass spectrometry. Microbial biomass carbon (MBC) was assessed via chloroform fumigation-extraction. Particulate organic carbon (POC) was isolated by sodium hexametaphosphate dispersion followed by wet sieving and quantified using the external-heat potassium dichromate oxidation method. Active organic carbon (AOC) was calculated as the sum of DOC, MBC, and ROC.

### 2.5 Soil aggregate stability analysis

Mechanically-Stable Aggregates (Dry Sieving Method) (Puchner, 1911; Díaz-Zorita et al., 2002): A 400 g air-dried soil sample was placed on a nest of sieves with mesh sizes of 5, 2, 1, 0.5, and 0.25 mm. Samples were shaken for 10 minutes at an amplitude of 2.0 mm using a vibratory sieve shaker (GRINDER SS200). The mass of soil retained on each sieve was measured. Water-Stable Aggregates (Wet Sieving Method) (Elliott, 1986): A 50 g subsample, proportionally representing the aggregate size fractions obtained from dry sieving, was placed on an identical nest of sieves. The sieve nest was submerged in deionized water within the bucket of a constant-temperature aggregate analyzer, ensuring the top sieve rim remained below the water level. After 10 min of immersion, oscillation commenced at a frequency of 30 oscillations min^-^¹ for 10 minutes. Aggregates retained on each sieve were collected, transferred to aluminum boxes, oven-dried, and weighed to determine the mass fraction of each aggregate size class.

Aggregate stability was characterized using four indices: MWD, GMD, PAD, and MA. MWD and GMD reflect water stability, with higher values indicating greater aggregate stability. Conversely, lower PAD values denote enhanced structural integrity. MA represents the structural framework of soil aggregates, exhibiting a positive correlation with soil fertility (Eynard et al., 2004). All indices were calculated as follows (Wang et al., 2019; Liu, 2015):


$$\text{MA}(\%)=\frac{\mathrm{Wma}}{\mathrm{Wss}}\times 100\%$$

$$\text{MWD(mm)}=\sum_{i=1}^{n}XiWi$$

$$\text{GMD(mm)}=Exp(\sum_{i=1}^{n}Wi\text{ ln}\bar{X}i)$$

$$\text{PAD(}\%)=\frac{\text{Wd}-\text{Ww}}{\text{Wd}}\times 100\%$$

In this study: W_ma_ refers to the mass of soil macroaggregates (>0.25 mm) (g); W_ss_ refers to the mass of the sieved soil sample (g); MWD refers to the mean weight diameter of aggregates (mm); GMD refers to the geometric mean diameter of aggregates (mm); X_i_ refers to the mean diameter of the i-th aggregate size fraction (mm); W_i_ refers to the mass percentage of the i-th aggregate size fraction (%); W_d_ refers to the mass fraction of dry-sieved aggregates retained on sieves with a mesh size >0.25 mm.

We used the area of the parameter-standardized radar chart as a comprehensive index to evaluate soil quality, with the calculation method as follows: Soil basic indicators were divided into two categories: “higher-is-better” and “lower-is-better”. A linear scoring function was adopted to standardize the soil indicators to scores between 0 and 1 (Bastida et al., 2006):


$$\text{SLi}=\frac{X}{X_{max}}$$

Where SLi is the linear score of the soil indicator, and X and Xmax are the measured value and maximum value of the soil indicator, respectively (Zhou et al., 2020).

The Soil Quality Index (SQI) was calculated based on SLi (Kuzyakov et al., 2020):


$$\text{SQI}=0.5\times \sum_{1}^{n}SLi^{2}\times \sin\left(\frac{2\pi }{n}\right)$$

Where n is the number of indicators.

Nitrogen Recovery Efficiency (REN, %), Agronomic Nitrogen Efficiency (AEN, %), and Partial Factor Productivity of Nitrogen Fertilizer (PFPN) were calculated using the following formulas, referring to the method described by (Wei et al., 2024):


$$\text{PFPN}=\frac{GY_{with\;N}}{NR}$$

$$\text{AEN}=\frac{\text{G}{\text{Y}}_{\text{with}\;\text{N}}-\text{G}{\text{Y}}_{\text{without}\;\text{N}}}{\text{NR}}\times 100\%$$

$$\text{REN}=\frac{\text{N}{\text{A}}_{\text{with}\;\text{N}}-\text{N}{\text{A}}_{\text{without}\;\text{N}}}{\text{NR}}\times 100\%$$

NA_with N_ and NA_without N_ refer to the plant nitrogen accumulation in the nitrogen-fertilized plot and non-nitrogen-fertilized plot, respectively; NR is the total annual nitrogen application rate; GY_with N_ and GY_without N_ refer to the grain yield in the nitrogen-fertilized plot and non-nitrogen-fertilized plot, respectively.

### 2.6 Soil enzymatic activities

Activities of soil enzymes—superoxide dismutase (SOD), catalase (CAT), peroxidase (POD), polyphenol oxidase (PPO), cellulase (CL), and β-Glucosidase—were assayed using commercial kits with detection by visible spectrophotometry (Saiya-Cork et al., 2002).

The soil Enzyme Multifunctionality (EMF) was derived from six soil enzyme activities (SOD, CAT, POD, βG, CL, and PPO). The soil multifunctionality index was obtained by standardizing the soil enzyme activities using Z-score standardization:


Z−score=(x meani)/SDi

where x is the measured enzymatic activity, the mean is the average of soil enzyme i, and SD is the standard deviation of soil enzyme i.EMF is the arithmetic mean of the Z-scores of each enzyme (Delgado-Baquerizo et al., 2016).

### 2.7 Statistical analyses

The data were analyzed by SPSS Statistics 20 software (SPSS Inc., Chicago, IL, USA). Based on the research objectives of different treatments, a split-plot analysis strategy with a randomized complete block design (RCBD) was adopted. Significant differences were determined by the least significant difference (LSD) method (P< 0.05). Analysis of variance (ANOVA) was used to assess the main and interactive effects of year, green manure, and biochar on maize yield and soil physicochemical properties under 100% nitrogen application (T1-T4). Pearson correlation analysis was applied to explore the relationships between soil carbon components, aggregate stability indices, and enzyme activities. Linear regression analysis was performed to examine the linear correlation between the soil quality index (SQI) and ecosystem multifunctionality (EMF), as well as between SQI and maize grain yield. All figures were generated using Origin 2021 software (Origin Lab Corp., Northampton, MA, USA).

## 3 Result

### 3.1 Grain yield, nitrogen accumulation, and nitrogen uptake efficiency of maize

**Figure 1:**
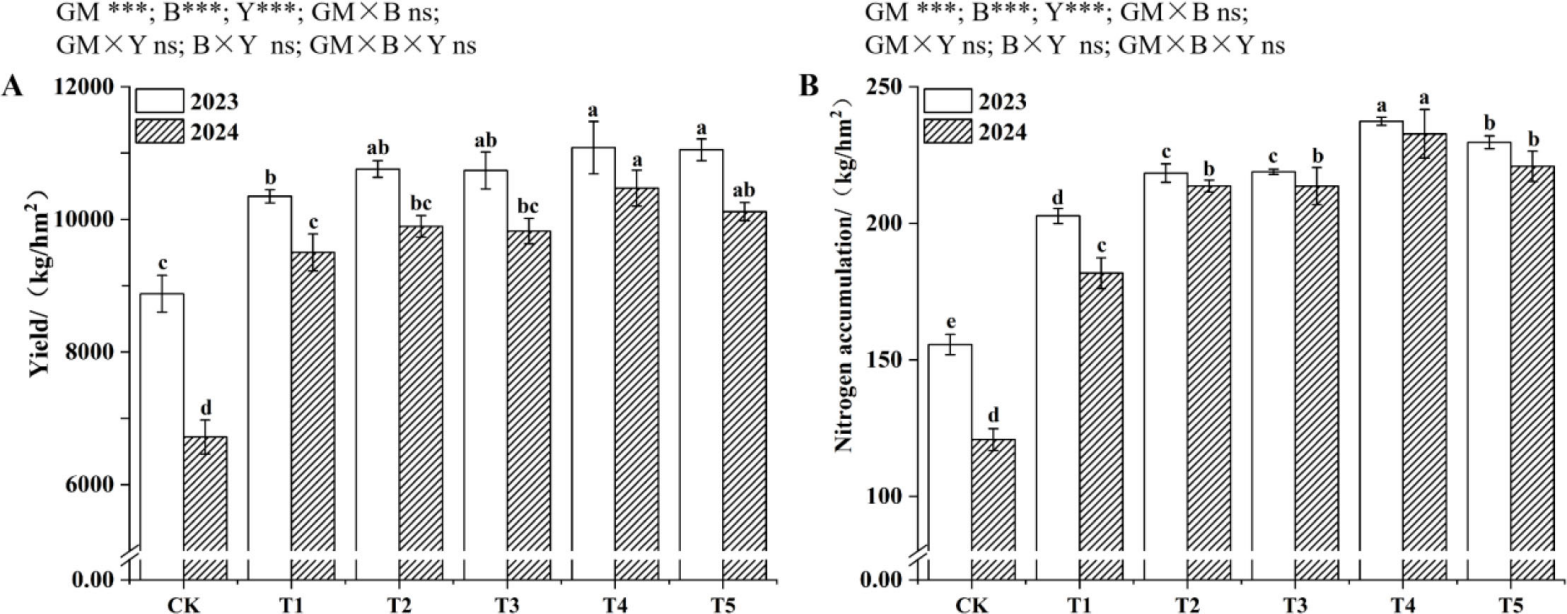
Yield of maize under different treatments **(A)**; Nutrient accumulation of maize under different treatments **(B)**. Data are presented as mean ± SE (n=3). Different lowercase letters indicate significant differences between different treatments in the same year (*P*<0.05; LSD). Asterisks indicate significance levels (*p< 0.05, **p< 0.01, ***p< 0.001; ANOVA). ns = non-significant.

As shown in **Figure 1**, the maize grain yield and nitrogen accumulation in 2024 were generally lower than those in 2023, but the variation trends among different treatments within each year were consistent. Over the two years, both grain yield and nitrogen accumulation were the highest in T4, followed by T5; T2 and T3 had higher values than T1, with T2 numerically higher than T3 in terms of grain yield. Compared with CK, the nitrogen application increased maize grain yield and nitrogen accumulation to varying degrees. Both biochar and green manure application alone could improve maize grain yield and nitrogen accumulation, while the combined application of green manure and biochar showed a better effect. Moreover, under the 80% nitrogen, the combined application could still maintain or increase maize grain yield and nitrogen accumulation.

As shown in **Table 1**, the nitrogen uptake efficiency of maize varied among different treatments at different times. In both 2023 and 2024, the Agronomic Nitrogen Efficiency (AEN), Partial Factor Productivity of Nitrogen Fertilizer (PFPN), and Nitrogen Recovery Efficiency (REN) of maize were the lowest in T1 and the highest in T5. Compared with other treatments, T5 increased AEN by 2.84~6.92 kg/kg and 2.75~8.14 kg/kg, PFPN by 15.17~19.25 kg/kg and 12.08~17.47 kg/kg, and REN by 6.01~25.25 percentage points and 7.28~35.62 percentage points in 2023 and 2024, respectively. These results indicate that after the combined application of biochar and green manure, the nitrogen use efficiency under both conventional nitrogen application and 80% nitrogen treatments was higher than that under the individual application of biochar or green manure.

**Table 1:** Nitrogen utilization rate of maize under different treatments.

Treatment	AE_N_ (kg/kg)		PFPN (kg/kg)		RE_N_ (%)	
	2023	2024	2023	2024	2023	2024
CK	—	—	—	—	—	—
T_1_	8.17 ± 1.71 c	15.46 ± 2.11 b	57.49 ± 0.55 c	52.79 ± 1.55 c	26.18 ± 2.38 d	33.87 ± 1.46 d
T_2_	10.45 ± 1.88 bc	17.65 ± 1.77 b	59.77 ± 0.69 bc	54.97 ± 0.91 c	34.88 ± 1.14 c	51.60 ± 1.21 c
T_3_	10.33 ± 0.59 bc	17.25 ± 0.61 b	59.65 ± 1.55 bc	54.57 ± 1.07 c	35.16 ± 1.53 c	51.59 ± 2.48 c
T_4_	12.25 ± 1.16 b	20.85 ± 1.47 a	61.57 ± 2.18 b	58.18 ± 1.51 b	45.42 ± 2.90 b	62.21 ± 2.96 b
T_5_	15.09 ± 1.23 a	23.60 ± 2.07 a	76.74 ± 1.14 a	70.26 ± 0.94 a	51.43 ± 1.79 a	69.49 ± 1.85 a
Statistical parameters for ANOVA:						
GM	4.876*		17.847***		200.201***	
B	0.533		22.248***		195.762***	
Y	140.779***		106.228***			
GM×B	1.207		0.246		2.686	
GM×Y	28.447***		0.234		6.779*	
B×Y	6.687*		0.56		7.712*	
GM×B×Y	1.932		0.713		6.563*	

Data are presented as mean ± SE (n=3). Different lowercase letters indicate significant differences between different treatments in the same year (*P*<0.05; LSD). Asterisks indicate significance levels (* p< 0.05, **p< 0.01, ***p< 0.001; ANOVA).

### 3.2 Nutrients and carbon fractions

As shown in **Table 2**, the soil nutrient contents varied among different treatments. Compared with CK, nitrogen application (T1) increased the contents of soil organic matter (OM), total nitrogen (TN), available phosphorus (AP), and available potassium (AK). After the individual application of biochar and green manure (T2 and T3), the soil pH, OM, TN, AP, and AK contents showed an increasing trend compared with T1. The combined application of biochar and green manure (T4) resulted in the highest soil pH, OM, TN, AP, and AK contents among all treatments. When nitrogen was applied at 80% (T5), the soil nutrient contents were slightly lower than those in T4 but higher than those in T2 and T3. Overall, the soil pH, OM, TN, AP, and AK contents followed the order as T4 > T5 > T2 ≈ T3 > T1 > CK; there were no significant differences in soil pH among all treatments.

**Table 2:** Soil nutrient content under different treatments.

Treatment	pH	OM (g/kg)	TN (g/kg)	AP (mg/kg)	AK (mg/kg)
CK	6.63 ± 0.07 a	42.60 ± 1.34 c	1.91 ± 0.02 c	35.90 ± 1.76 b	220.66 ± 7.42 c
T1	6.69 ± 0.02 a	44.54 ± 3.38 c	2.04 ± 0.08 c	36.71 ± 1.29 b	223.25 ± 7.03 c
T2	6.73 ± 0.07 a	52.51 ± 1.60 b	2.34 ± 0.11 b	37.50 ± 1.50 b	231.67 ± 8.66 bc
T3	6.78 ± 0.14 a	52.46 ± 2.23 b	2.31 ± 0.11 b	41.35 ± 3.61 a	243.91 ± 10.55 ab
T4	6.80 ± 0.08 a	59.05 ± 3.71 a	2.50 ± 0.02 a	43.20 ± 2.34 a	249.55 ± 6.10 a
T5	6.78 ± 0.22 a	58.06 ± 3.85 a	2.45 ± 0.06 ab	42.82 ± 1.56 a	246.22 ± 7.63 a
Statistical parameters for ANOVA:					
GM	1.181	19.052***	23.367***	17.201***	17.308***
B	0.21	19.301***	30.879***	1.128	2.302
GM*B	0.015	0.176	1.742	0.18	0.09

Asterisks indicate significance levels (*p< 0.05, **p< 0.01, ***p< 0.001, ns = non-significant).

As shown in **Table 3**, the soil carbon fractions differed among different treatments. Compared with CK, nitrogen application increased the soil carbon fractions. The soil carbon fractions in T2 and T3 (separate application of biochar and green manure) were significantly higher than those in T1. The contents of total carbon (TC), organic carbon (OC), readily oxidizable carbon (ROC), dissolved organic carbon (DOC), microbial biomass carbon (MBC), active organic carbon (AOC), and particulate organic carbon (POC) in T4 (combined application of biochar and green manure) were higher than those in other treatments. When nitrogen application was applied at 80% (T5), the soil carbon fractions were lower than those in T4 but higher than those in T2 and T3.

**Table 3:** Effects of treatments on carbon fractions.

Treatment	TC (g/kg)	OC (g/kg)	ROC (g/kg)	DOC (mg/kg)	MBC (mg/kg)	AOC (%)	POC (mg/kg)
CK	25.05 ± 1.76 d	24.71 ± 0.78 c	2.02 ± 0.06 d	95.40 ± 5.23 c	413.62 ± 15.56 c	25.31 ± 0.64 d	4.55 ± 0.24 b
T1	25.97 ± 2.74 cd	25.83 ± 1.96 c	2.16 ± 0.17 cd	103.75 ± 7.88 bc	440.26 ± 18.27 bc	27.05 ± 1.81 cd	5.09 ± 0.08 a
T2	29.49 ± 1.83 bc	30.46 ± 0.93 bc	2.39 ± 0.14 bcd	105.47 ± 2.32 bc	458.28 ± 17.91 b	29.54 ± 1.34 bcd	5.11 ± 0.18 a
T3	30.53 ± 1.67 ab	30.43 ± 1.30 ab	2.57 ± 0.11 abc	104.16 ± 2.80 bc	446.40 ± 12.01 b	31.23 ± 1.12 abc	5.16 ± 0.07 a
T4	33.50 ± 2.85 a	34.25 ± 2.15 a	2.93 ± 0.41 a	116.89 ± 5.19 a	507.77 ± 18.18 a	35.55 ± 4.32 a	5.40 ± 0.43 a
T5	33.05 ± 1.13 ab	33.68 ± 2.24 a	2.80 ± 0.44 ab	108.26 ± 8.68 ab	501.66 ± 19.51 a	34.19 ± 4.35 ab	5.23 ± 0.11 a
Statistical parameters for ANOVA:							
GM	12.628**	19.052***	9.608**	3.067	7.95*	10.419**	1.915
B	7.236*	19.301***	3.659	4.582	16.189**	4.654	0.981
GM*B	0.051	0.176	0.173	2.659	4.828*	0.332	0.78

Data are presented as mean ± SE (n=3). Different lowercase letters indicate significant differences between different treatments in the same year (*P*<0.05; LSD). Asterisks indicate significance levels (* p< 0.05, **p< 0.01, ***p< 0.001; ANOVA).

### 3.3 Soil aggregate distribution and aggregate stability

**Figure 2:**
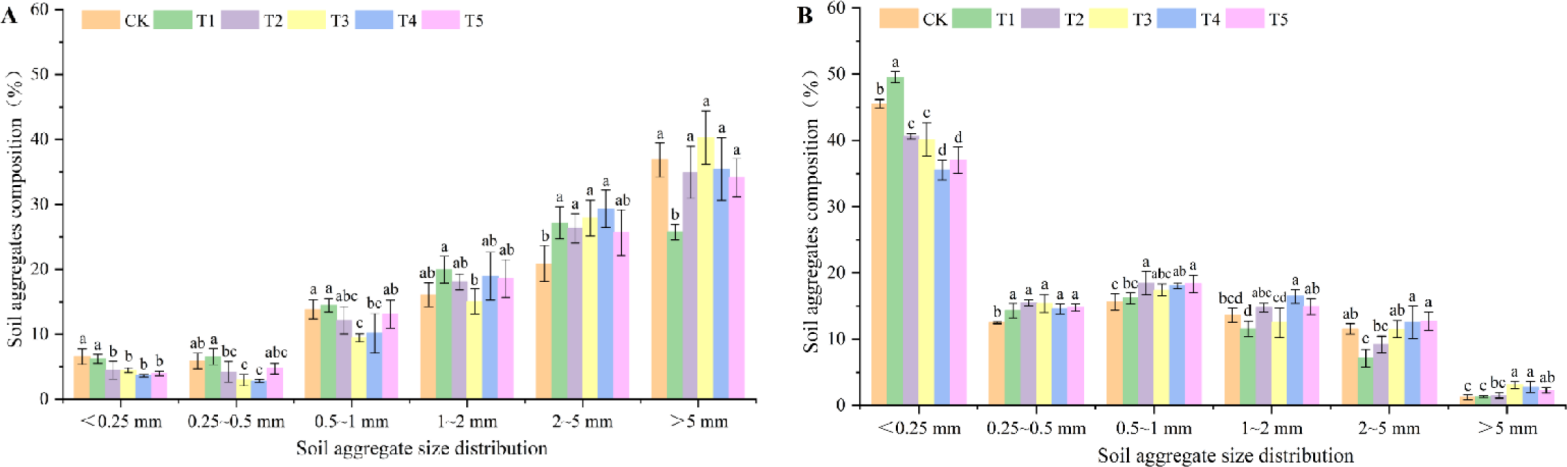
Effects of different treatments on composition and distribution of soil mechanical stability **(A)** and water stable aggregate **(B)**. Data are presented as mean ± SE (n = 3). Different lowercase letters indicate significant differences between different treatments in the same year (P<0.05).

The characteristics of soil mechanically stable aggregates (MSAs) and water-stable aggregates (WSAs) under different treatments are shown in **Figure 2**, with opposite particle size distribution trends between the two. For MSAs, the content increased with increasing particle size: compared with CK, all treatments reduced the content of aggregates< 0.25 mm, among which T4 showed the highest reduction (45.04%), and correspondingly increased the proportion of aggregates between 0.25~5 mm. Compared with T1, the treatments with biochar and/or green manure application (T2~T5) further reduced the content of aggregates< 0.25 mm and increased the proportion of aggregates > 5 mm. For WSAs, the content decreased with increasing particle size: compared with T1, T2~T5 treatments significantly reduced the content of aggregates< 0.25 mm, with T4 showing the highest reduction (28.29%), and simultaneously increased the proportion of aggregates between 0.25~5 mm.

As shown in **Table 4**, the soil aggregate stability varied among different treatments. Regarding the mechanical stability indices: the mechanically stable mean weight diameter (MS-MWD) was the lowest in T1, and the MS-MWD values under treatments with biochar and/or green manure application were 0.32~0.63 mm higher than that in T1; the mechanically stable geometric mean diameter (MS-GMD) was the highest in T3, but there was no significant difference between T3 and T2/T4. The aggregate distribution of all treatments was dominated by mechanically stable macroaggregates (MS-MA), accounting for over 90% of the total aggregates, with T4 showing the highest proportion (96.59%). Regarding the water stability indices: the water-stable mean weight diameter (WS-MWD) was the lowest in T1 (0.12~0.32 mm lower than other treatments), and the WS-MWD values under treatments with biochar and/or green manure application were 0.13~0.32 mm higher than that in T1; the water-stable geometric mean diameter (WS-GMD) and water-stable macroaggregates (WS-MA) were the highest in T4, but there was no significant difference between T4 and T5. For the percentage of aggregate destruction (PAD), T4 showed the smallest value, which was significantly lower than that of other treatments by 4.11~12.95 percentage points, except for no significant difference with T5.

**Table 4:** Effects of different treatments on MWD, GMD, MA and PAD of soil aggregates.

处理 Treatment	Mechanically stable index			Water stable index			PAD/(%)
	MS-MWD (mm)	MS-GMD (mm)	MS-MA (%)	WS-MWD (mm)	WS-GMD (mm)	WS-MA (%)	
CK	2.96 ± 0.09 bc	2.08 ± 0.12 cd	93.43 ± 1.20 b	0.91 ± 0.02 b	0.56 ± 0.01 c	54.45 ± 0.63 c	41.72 ± 1.18 b
T1	2.68 ± 0.07 c	1.90 ± 0.07 d	93.80 ± 0.67 b	0.79 ± 0.05 c	0.49 ± 0.02 d	50.47 ± 0.85 d	46.19 ± 1.21 a
T2	3.05 ± 0.23 ab	2.30 ± 0.28 abc	95.55 ± 1.36 a	0.92 ± 0.04 b	0.57 ± 0.01 c	59.41 ± 0.39 b	37.81 ± 1.00 c
T3	3.31 ± 0.09 a	2.56 ± 0.06 a	95.57 ± 0.36 a	1.03 ± 0.06 a	0.60 ± 0.03 bc	59.87 ± 2.50 b	37.35 ± 2.39 cd
T4	3.18 ± 0.21 ab	2.49 ± 0.22 ab	96.59 ± 0.45 a	1.11 ± 0.05 a	0.66 ± 0.03 a	64.48 ± 1.50 a	33.24 ± 1.69 e
T5	3.01 ± 0.15 a	2.24 ± 0.14 bc	96.05 ± 0.40 a	1.07 ± 0.02 a	0.63 ± 0.02 ab	62.99 ± 1.99 a	34.42 ± 2.34 de
Statistical parameters for ANOVA:							
GM	20.023***	17.223***	8.298*	76.507***	66.086***	63.872***	47.915***
B	2.793	1.769	8.173*	16.428**	30.15***	56.038***	41.594***
GM*B	6.235*	7.781*	0.557	1.242	0.525	5.749*	4.858*

Data are presented as mean ± SE (n=3).Different lowercase letters indicate significant differences between different treatments in the same year (*P*<0.05; LSD). Asterisks indicate significance levels (* p< 0.05, **p< 0.01, ***p< 0.001; ANOVA).

### 3.4 Differences in soil enzyme activities

As shown in **Table 5**, the soil enzyme activities varied among different treatments. Compared with CK, nitrogen application increased soil enzyme activities, with significantly higher activities of SOD, CAT, POD, PPO, CL, andβG in T1. After the separate application of biochar and green manure, the soil enzyme activities in T2 and T3 showed a significant increasing trend compared with T1. Following the combined application of biochar and green manure, the activities of SOD, CAT, POD, PPO, cellulase, and β-glucosidase in T4 were higher than those in other treatments. When nitrogen application was reduced by 20%, the soil enzyme activities in T5 were lower than those in T4 but still higher than those in T2 and T3.

**Table 5:** Effects of different treatments on soil enzyme activities.

Treatment	SOD (U/g)	CAT (μmol/d/g)	POD (mg/d/g)	PPO (mg/d/g)	Cellulase (mg/d/g)	β-Glucosidase (μmol/d/g)
CK	228.91 ± 5.34 d	32.47 ± 1.74b	20.87 ± 2.75 b	27.44 ± 1.21 d	222.21 ± 7.13 b	36.04 ± 2.40 c
T1	242.80 ± 6.26 c	33.24 ± 1.61b	21.01 ± 0.94 b	29.77 ± 1.88 c	231.36 ± 8.76 b	44.23 ± 1.61 b
T2	276.19 ± 6.69 b	33.84 ± 0.65b	22.65 ± 1.39 ab	31.00 ± 0.59 bc	247.15 ± 7.51 a	47.18 ± 2.10 ab
T3	272.69 ± 8.54 b	34.11 ± 0.85b	22.43 ± 1.90 ab	31.23 ± 1.25 bc	249.45 ± 5.69 a	46.75 ± 3.48 ab
T4	292.06 ± 7.55 a	37.31 ± 1.28a	24.17 ± 1.33 a	33.60 ± 1.45 a	258.22 ± 5.41 a	50.18 ± 2.08 a
T5	284.19 ± 6.44 ab	37.07 ± 0.38a	22.35 ± 1.11 ab	33.05 ± 1.11 ab	254.14 ± 4.94 a	48.93 ± 1.92 a
Statistical parameters for ANOVA:						
GM	33.168***	9.912**	2.293	7.219*	14.169**	4.174
B	44.104***	7.65*	3.035	5.695*	10.052**	5.587*
GM*B	3.114	3.552	0.002	0.578	0.82	0.032

Data are presented as mean ± SE (n=3). Different lowercase letters indicate significant differences between different treatments in the same year (*P*<0.05; LSD). Asterisks indicate significance levels (* p< 0.05, **p< 0.01, ***p< 0.001; ANOVA).

### 3.5 Correlation analysis of SQI, EMF, and GY

**Figure 3:**
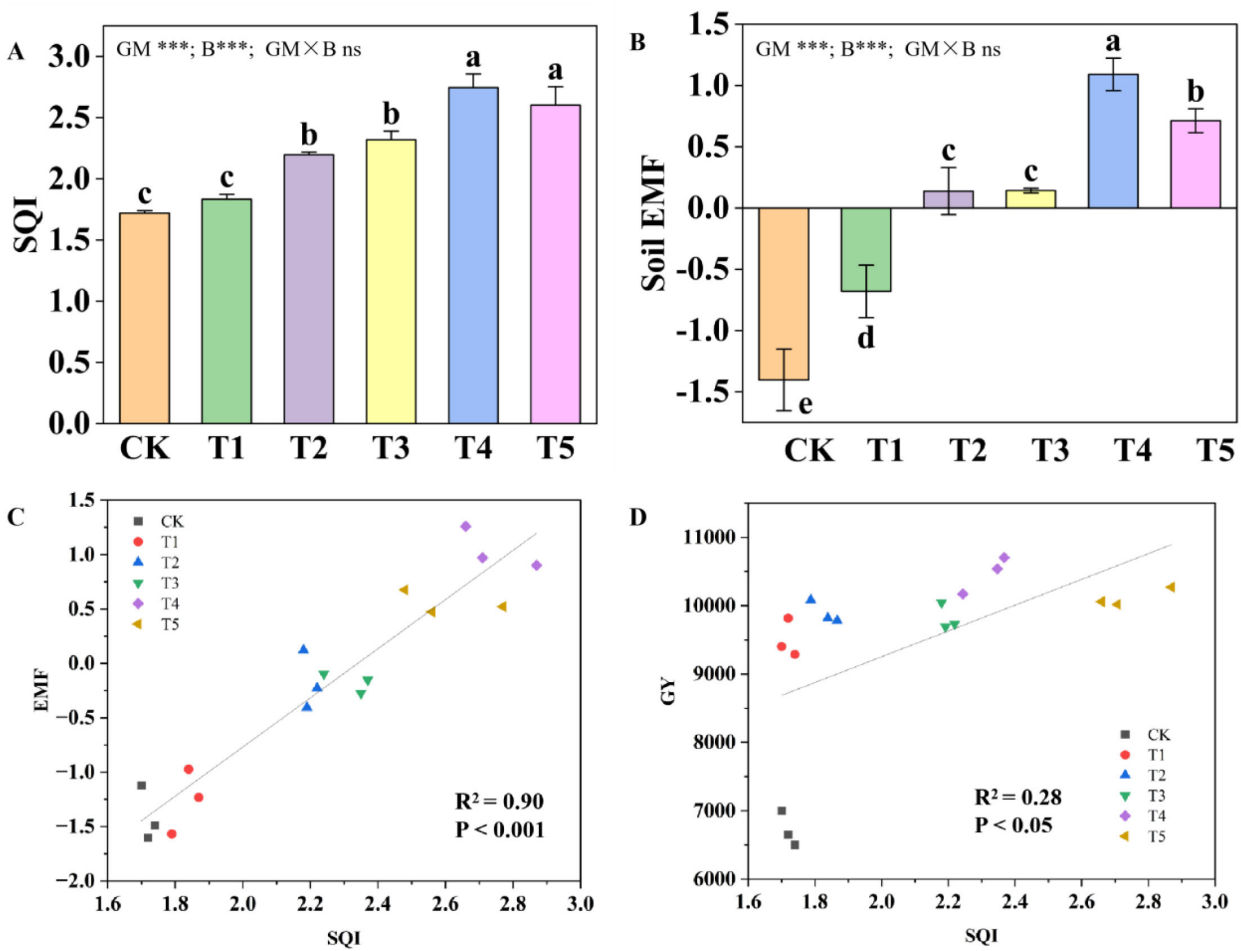
Responses of **(A)** Soil Quality Index (SQI) and **(B)** soil EMF to different treatments. **(C)** Relationship between soil EMF and SQI analyzed by linear regression. **(D)** Relationship between Grain Yield (GY) and SQI analyzed by linear regression. Data are presented as mean ± SE (n=3). Different lowercase letters indicate significant differences between different treatments in the same year (P<0.05; LSD). Asterisks indicate significance levels (* p< 0.05, **p< 0.01, ***p< 0.001; ANOVA).

Compared with CK, treatments T2, T3, T4, and T5 all significantly increased the SQI, with T4 showing the greatest increase (49.78%). Similarly, all treatments significantly enhanced soil EMF compared with CK, with the increment ranging from 0.723 to 2.494 units in the order of T4 > T5 > T2 ≈ T3 > T1 (**Figures 3A, B**). SQI exhibited a significant positive linear correlation with soil EMF (**Figure 3C**), and a moderate positive linear correlation with Grain Yield (GY). These results indicate that the combined application of green manure and biochar synergistically enhances the improvement effects of both SQI and EMF, which persist even under nitrogen reduction conditions, and further influences maize grain yield through SQI.

### 3.6 Analysis of correlations between carbon fractions and aggregates, enzyme activities, and their influencing factors under different treatments

**Figure 4:**
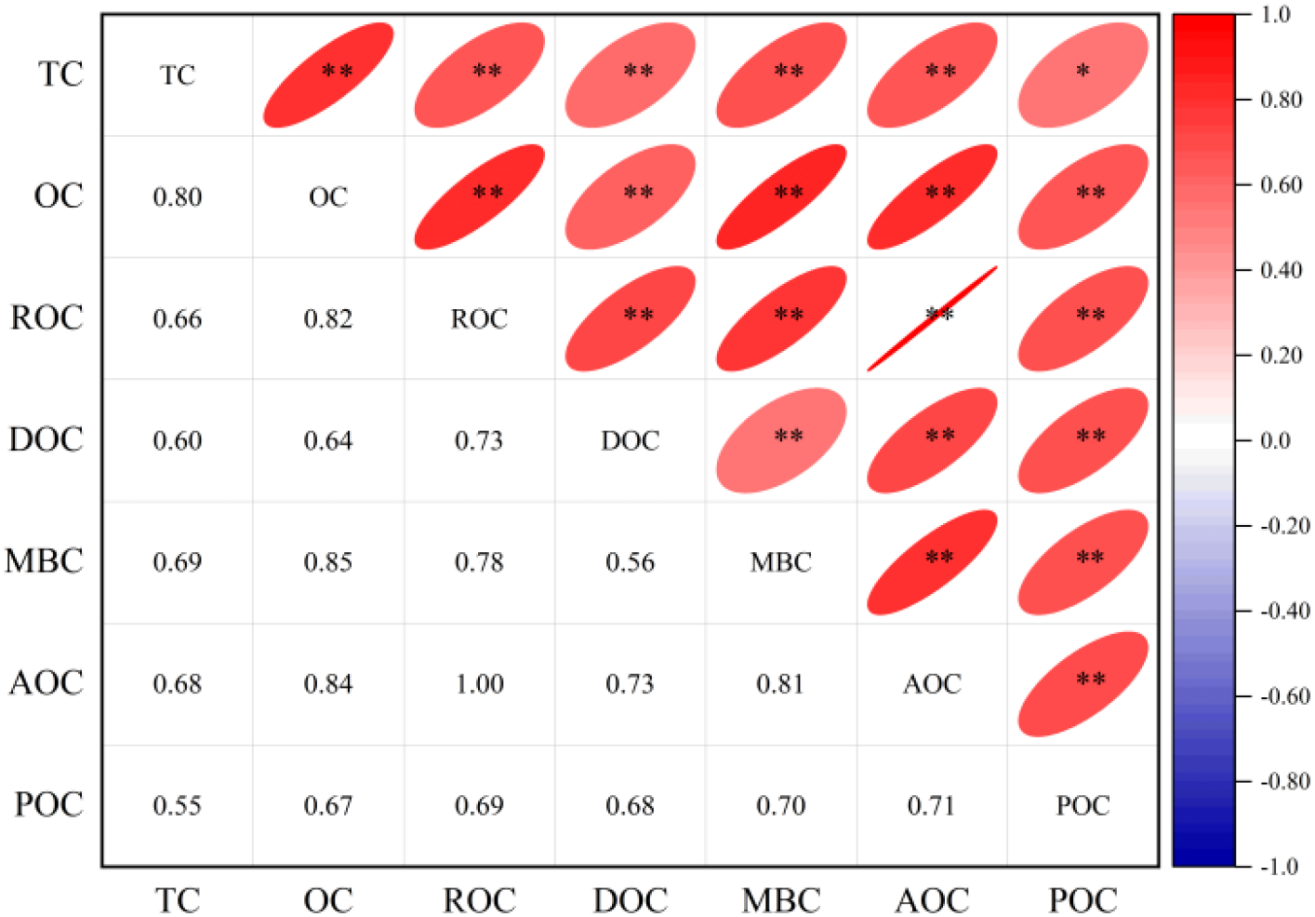
Correlation analysis of soil carbon components. ** and * indicate extremely significant and significant correlation at 0.01 and 0.05 level, respectively.

As shown in **Figure 4**, correlation analysis revealed significant to highly significant positive correlations among soil carbon fractions. TC exhibited highly significant positive correlations with OC, ROC, DOC, MBC, and AOC, while showing only a significant positive correlation with POC. All other carbon fractions demonstrated highly significant positive intercorrelations.

**Figure 5:**
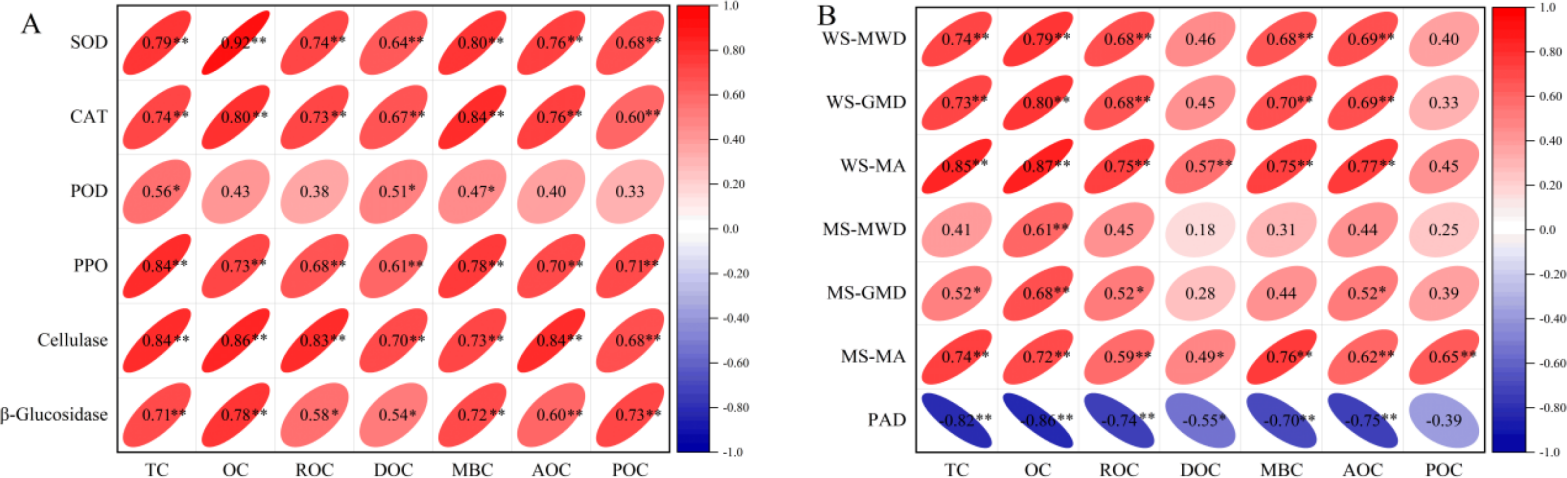
Correlation between soil carbon components and aggregate stability **(A)** and enzyme activity **(B)**.

Correlation analysis between soil carbon fractions and aggregate stability indices, as well as enzyme activities, revealed significant relationships (**Figure 5**). Soil carbon fractions exhibited highly significant positive correlations with SOD, CAT, PPO, and cellulase. While POC, DOC, and MBC showed significant positive correlations with POD, other carbon fractions displayed no significant correlation with POD. ROC and DOC demonstrated significant positive correlations with β-glucosidase, whereas all other carbon fractions exhibited highly significant positive correlations with this enzyme.

All carbon fractions showed highly significant positive correlations with MS-MA. Except for DOC and POC—which showed no correlation with WS-MWD and WS-GMD —other carbon fractions had significant to highly significant positive correlations with these indices. Similarly, all carbon fractions except POC showed significant to highly significant positive correlations with WS-MA, while POC exhibited no correlation. OC showed a highly significant positive correlation with MS-MWD, but other carbon fractions showed no significant relationship. Most carbon fractions demonstrated significant to highly significant positive correlations with MS-GMD, except DOC, MBC, and POC which showed no correlation. Carbon fractions exhibited significant to highly significant negative correlations with PAD, except POC which showed no correlation. Crucially, correlations between carbon fractions and water-stable aggregate indices were stronger than those with mechanical stability indices. Collectively, soil carbon fractions showed strong correlations with enzyme activities and water-stable aggregate indices. Increased carbon fractions corresponded with larger water-stable aggregates, reduced PAD values, and enhanced soil structural stability.

## 4 Discussion

Soil serves as the base of agricultural practices, and its quality directly influences both crop yield and quality, thereby influencing food security (Zhang et al., 2025). The soil quality index (SQI) is a critical indicator of soil fertility and ecological function (Lehmann and Kleber, 2015), exerting significant influences on nutrient availability, productivity, and structural stability (Qu et al., 2022), which may significantly affect yield formation (Zhang et al., 2026). The present study demonstrated that 100% nitrogen application significantly increased the contents of soil organic matter (SOM), total nitrogen (TN), available phosphorus (AP), and available potassium (AK), compared with the control (0% N), which is consistent with previous findings on the efficacy of fertilizers in improving soil fertility (Meng et al., 2024). Relative to the individual nitrogen application (100% N), the individual application of biochar (100% NB) or green manure (100% NM) further elevated the contents of the aforementioned nutrients, confirming their important role as supplementary nutrient sources. Compared with individual applications, the combined application of green manure and biochar provides a broader range of resources and higher nutrient availability, increases the contents of soil carbon fractions (**Table 3**), and thereby effectively enhances the physical and chemical properties of the soil. This interrelationship indicates the coupling of soil nutrient cycling and carbon sequestration. In a recent study by Sun et al. (2023) also reported that biochar blended with nitrogen fertilizer can improve the fertility and productivity of black soils in northeast China, while nitrogen decrease is possible and essential for maintaining grain yield. Under the condition of 80% nitrogen application (T4), the soil nutrient levels were still higher than those under the individual nitrogen (100% N), green manure (100% NM), and biochar application (100% NB) treatments, suggesting that organic amendments can achieve partial nutrient substitution, which is consistent with prior research (Lochon et al., 2018).

The combined application of green manure and biochar increased soil enzyme activities and soil EMF (**Figure 3B**). Soil enzyme activities were highly correlated with various soil carbon fractions (**Figure 3**), indicating that soil enzymes improve nutrient availability for microorganisms and plants, while enzyme production is mediated by substrate availability. Ma et al. (2021) reported that nitrogen fertilizer inputs are relatively easily accessible, reducing nitrogen competition between microorganisms and crops. In another study, carbon accumulated by plant roots after direct nitrogen input stimulates nutrient cycling and similarly promotes microorganisms to produce new soil enzymes (Dong et al., 2021). Optimizing green manure and nitrogen enhances maize photosynthesis via enhanced soil properties and increasing yield (Wang et al., 2025). Zhang et al. (2023) also stated that adding Chinese milk vetch into the field along with a 20% reduction in nitrogen fertilizer did not lead to any reduction in early rice yield.

Soil aggregates are the basic units of soil structure and important carriers of soil fertility. Aggregate stability is a key determinant of soil quality, with higher stability indicating better soil conditions. This stability is typically quantified using indices such as mean weight diameter (MWD), geometric mean diameter (GMD), macroaggregate content (MA), and percentage of aggregate destruction (PAD) (Six et al., 2004). Our study found that compared with the individual nitrogen application treatment (100% N), the individual applications of biochar (100% NB) and green manure (100% NM), as well as their combined application (T5), all increased MWD, GMD, and MA while decreasing PAD. This confirms the effectiveness of biochar and green manure in improving soil structural stability—consistent with previous research findings on biochar and green manure (Zhao et al., 2024). Notably, for both mechanically stable and water-stable aggregates, the individual nitrogen application (100% N) exhibited lower MWD and GMD but higher PAD than the no-nitrogen control (0% N). This phenomenon may be attributed to nitrogen application-induced depletion of exchangeable cations (Yao et al., 2024) and a reduction in fungal biomass—fungi are critical for macroaggregate formation (Zhang et al., 2021). Guizhou yellow soils inherently have weak water and nutrient retention capacities; the combined effects of the aforementioned factors lead to the breakdown of macroaggregates into smaller particles, thereby reducing soil stability. Correlation analysis revealed significant correlations between soil carbon fractions and water-stable aggregate indices (WS-MWD, WS-GMD, WS-MA), PAD, and enzyme activities. This implies that increased rhizospheric nutrient input and improved aggregate stability create a favorable microenvironment for microbial activities, significantly enhancing microbial biomass and enzyme functions, and ultimately accelerating soil carbon mineralization (Li et al., 2023).

Soil carbon dynamics are influenced by organic amendments: green manure serves as a labile carbon source (Zhu et al., 2014), whereas biochar represents a stable carbon source (Zou et al., 2025). Their co-application demonstrates synergistic effects (Zhang et al., 2024), enhancing soil carbon sequestration. This study revealed increased carbon fractions under biochar-green manure co-application with 100% nitrogen (T5) or 80% nitrogen application (T4) compared to individual amendments, promoting carbon mineralization and stable carbon pool formation. Notably, microbial biomass carbon (MBC) was higher under green manure (100% NM) than biochar (100% NB), likely due to limited microbial utilization of biochar’s recalcitrant carbon (Li et al., 2025). Microorganisms drive soil carbon cycling through necromass accumulation (Wang et al., 2021) and exudates that stabilize organic matter via mineral association and aggregate formation. Bacteria, fungi, and archaea regulate carbon cycling through humification and mineralization processes (Trejos-Espeleta et al., 2024; Thotakuri et al., 2024). Thus, elucidating microbial community dynamics (bacteria/fungi/archaea) is essential to decipher carbon cycling mechanisms in yellow soils under co-application. Given the long-term nature of biochar-green manure effects on carbon-nitrogen cycling, multi-year field trials are imperative to quantify sustained “carbon-nitrogen synergy”—an ongoing objective of this research program.

## 5 Conclusions

The combined application of biochar and green manure can significantly improve soil quality and maize production efficiency by enhancing soil nutrient status, constructing stable carbon pools, optimizing aggregate structure, and boosting microbial activity. The combined application of biochar and green manure with 20% reduced nitrogen application (T5) can still maintain favorable soil quality and yield levels and achieve the optimal nitrogen use efficiency. Present findings contribute to the field by offering evidence-based guidance for integrated soil fertility management under reduced synthetic fertilizer inputs.

## Data Availability

The raw data supporting the conclusions of this article will be made available by the authors, without undue reservation.
