# Correction: Integrated biochar and green manure improve maize productivity and soil health under reduced nitrogen fertilization in yellow soil

**DOI:** 10.3389/fpls.2026.1861708

**Published:** 2026-04-29

**Authors:** Quanquan Wei, Pengfei Liu, Xiangyang Guo, Meng Zhang, Lingling Liu, Xiaofeng Gu, Lei Zhang, Yifei Zou, Yu Li, Muzammal Rehman, Jiulan Gou

**Affiliations:** 1 Institute of Soil and Fertilizer, Guizhou Academy of Agricultural Sciences, Guiyang, Guizhou, China; 2 Guizhou Observation Experimental Station of Farmland Preservation and Agricultural Environmental Sciences, Ministry of Agriculture, Guiyang, Guizhou, China; 3 Institute of Upland Crops, Guizhou Academy of Agricultural Sciences, Guiyang, China; 4 MOA Key Laboratory of Crop Ecophysiology and Farming System in the Middle Reaches of the Yangtze River, College of Plant Science and Technology, Huazhong Agricultural University, Wuhan, Hubei, China; 5 Rapeseed Research Institute, Guizhou Academy of Agricultural Sciences, Guiyang, Guizhou, China; 6 Yunnan Key Laboratory of Potato Biology, School of Life Science, Yunnan Normal University, Kunming, China

**Keywords:** biochar, carbon fractions, green manure, soil aggregates, yellow soil

In the **Funding** statement, an incorrect **Funding** number “QKHPSSYS[2025]035” was provided for “Guizhou Key Laboratory of Cultivated Land Quality”. The correct number is “QKHPZSYS[2025]035”.

The original version of this article has been updated.

